# Modified Models for Predicting Malignancy Using Ultrasound Characters Have High Accuracy in Thyroid Nodules With Small Size

**DOI:** 10.3389/fmolb.2021.752417

**Published:** 2021-11-26

**Authors:** Shan Jiang, Qingji Xie, Nan Li, Haizhen Chen, Xi Chen

**Affiliations:** ^1^ Department of Vascular Thyroid Surgery, Affiliated Union Hospital, Fujian Medical University, Fuzhou, China; ^2^ Department of Surgery, Ruijin Hospital Affiliated of Shanghai Jiaotong University School of Medicine, Shanghai, China

**Keywords:** diagnostic, ultrasound characteristics, malignancy of thyroid nodules, nodule size, logistic model

## Abstract

To assess the malignancy risk of thyroid nodules, ten ultrasound characteristics are suggested as key diagnostic markers. The European Thyroid Association Guidelines (EU-TIRADS) and 2015 American Thyroid Association Management Guidelines (2015ATA) are mainly used for ultrasound malignancy risk stratification, but both are less accurate and do not appropriatetly classify high risk patients in clinical examination. Previous studies focus on papillary thyroid carcinoma (PTC), but follicular thyroid carcinoma (FTC) and medullary thyroid carcinoma (MTC) remained to be characterized. Thus, this study aimed to determine the diagnostic accuracy and establish models using all ultrasound features including the nodule size for predicting the malignancy of thyroid nodules (PTC, FTC, and MTC) in China. We applied logistic regression to the data of 1,500 patients who received medical treatment in Shanghai and Fujian. Ultrasound features including taller-than-wide shape and invasion of the thyroid capsule showed high odds ratio (OR 19.329 and 4.672) for PTC in this dataset. Invasion of the thyroid also showed the highest odds ratio (OR = 8.10) for MTC. For FTC, the halo sign has the highest odds ratio (OR = 13.40). Four ultrasound features revealed distinct OR in PTC nodule groups with different sizes. In this study, we constructed a logistic model with accuracy up to 80%. In addition, this model revealed more accuracy than TIRADS in 4b and 4c category nodules. Hence, this model could well predict malignancy in small nodules and classify high-risk patients.

## Introduction

Thyroid cancer, including papillary thyroid carcinoma (PTC), follicular thyroid carcinoma (FTC), and medullary thyroid carcinoma (MTC), is one of the least painful carcinoma, which develops into a solid malignant tumor. In recent years, the incidence and detection of thyroid carcinoma has been on the rise worldwide. Over the past 20 years, thyroid carcinoma has shown higher incidence rates, surpassing those of breast carcinoma and lung carcinoma (Vigneri et al., 2015; Seib and Sosa, 2019). The malignant rate of the thyroid nodule is more than 10% (Brito et al., 2014; Al Nofal et al., 2016; Singh Ospina et al., 2016). At present, clinical guidelines suggest that all patients should have ultrasound examination and combine with clinical factors to determine further validation tests, such as fine-needle aspiration biopsy (FNAB). As ultrasound assessment has wide availability, is not complex, and does not involve exposure to ionizing radiation, it has become a key diagnostic step to assess the risk of carcinoma in patients (Singh Ospina et al., 2016).

Research studies conducted in the United States have reported an association between the risk of malignancy and the following features: hypo-echogenicity (ECHO), taller-than-wide (TTW) shape, irregular edge (IRE), echogenic focus (EF), and invasion of the thyroid capsule (ITC) (Wolinski et al., 2014; Chambara and Ying, 2019). Nevertheless, none of these characteristics can be used as a single reliable factor to identify malignancies of the thyroid (Brito et al., 2014; Al Nofal et al., 2016; Singh Ospina et al., 2016). Brito et al. identified TTW (11.1; 95%CI: 6.6–18.9) and internal calcifications (6.8; 95%CI: 4.5–10.2) as the ultrasound features with the highest diagnostic odds ratio (OR). Campanelle et al. found that the top diagnostic OR belongs to TTW, absent halo sign, EF, and IRE. Remonti et al. revealed that TTW, EF, and absence of elasticity have the highest OR. Recently, Wettasinghe et al. suggested that EF, IRE, and ECHO are typical for diagnostic OR. Not only diagnostic OR, the specificity and sensitivity of these ultrasound parameters are variable in different studies. However, PTC accounted for more than 90% of all thyroid malignant tumors, and these data almost indicate risk factors for PTC. Other ultrasound features such as the halo sign (AUR) and location were minimally used as risk factors in clinical studies. Actually, other rare types of thyroid cancer can also have regional lymph node metastasis at the same time of blood metastasis, such as FTC and MTC. It was suggested that the evaluation of thyroid nodules based on rare types of cancer is also an important process of diagnosis and treatment of thyroid cancer. These topics still remained to be studied.

Thus, the diagnostic accuracy of these ultrasound features is not high. No significant evidence currently exists for any single characteristic. Clinically, it usually defines the risk according to the number of ultrasound features, which has great uncertainty (Oliveira et al., 2018). The European Thyroid Association Guidelines for ultrasound malignancy risk stratification (EU-TIRADS) suggested that the numbers of high-risk features including ECHO, TTW, IRE, EF, and ITC from 1 to > −4 could indicate the risk form 5 to 80%. The weight factor of each ultrasound feature and the combination pattern are not considered in US-TIRADS, which could play a critical role in the prediction of PTC. The 2015 American Thyroid Association Management Guidelines (2015ATA) suggested that nodules with high suspicion of malignancy (70–90%) has ECHO with one or more features, including IRE, MCAL, and TTW. The intermediate suspicion of malignancy in 2015ATA is 10–20%, where nodules only have ECHO. According to US-TIRADS and 2015ATA, recommended FNABs in 4b type of nodules only have one or more ultrasound feature than 4a. Also, recommended surgical operations in 4c type of nodules only have one or more ultrasound feature that 4b. However, which features among the five factors were not considered to validate the risk is not known. An alternative approach to accurately predict malignancy of thyroid nodules is building a mode to count the probability based on all five ultrasound features.

Moreover, the ultrasound assessment is still not solid and convincing to predict malignancy of thyroid nodules. FNAB was considered as the most conclusive method in clinical examination, whereas patients are not fully accepted to FNAB before carcinoma is finally determined. Even FNAB has limitations in clinical implementation. The nodule size, location, texture, and other factors will restrict the operation process of FANB. Importantly, when the nodule is less than 5 mm, FANB is more difficult due to the limitation of the puncture tool. Finally, these factors will reduce the accuracy of puncture. Thus, improvement of ultrasound assessment using typical ultrasound characteristics together with a nodule size is valuable in the current stage.

Thus, the present study aimed to establish models to finely explain the probability of thyroid nodules malignancy (PTC, FTC, and MTC) using currently revealed ultrasound features with high risk and nodule size. The analysis of more characteristics and prediction of the probability of malignant thyroid nodules to avoid confirmatory experiments will have an important impact on the guidelines and clinical recommendations.

### Patients and Methods

#### Patients and Data Collection

The study was reviewed and approved by the Ethics Committee of Fujian Medical University and the Ethics Committee of Ruijin Hospital Affiliated to the Shanghai Jiao Tong University Medical School. From January 2015 to August 2019, 1,505 consecutive patients (with clinical diagnosis) at Ruijin Hospital and Fujian Union Hospital were included in the study. Among them, 385 had benign tumors, 1,000 had PTC, 80 had FTC, and 40 had MTC. The malignant thyroid carcinoma was confirmed as PTC, FTC, or MTC by pathologic diagnosis after thyroidectomy. The benign cases were confirmed by FNAB or pathologic diagnosis after thyroidectomy. We randomly selected 500 PTC and 193 benign cases for developing the prediction model, and 500 PTC and 192 benign cases for validation. All FTC or MTC cases were used for developing the prediction model.

Ultrasound scanning was performed by several radiologists from two hospitals, who have many years of experience in thyroid ultrasound scanning. Ten ultrasound characteristics of the thyroid nodules in all patients were assessed by the radiologist, including 1) hypo-echogenicity (ECHO); 2) irregular or micro-lobulated margins (IRE); 3) taller-than-wide ratio >1 (TTW); 4) echogenic focus (EF); 5) invasion of the thyroid capsule (ITC); 6) blood flow by color Doppler flow imaging (CDFI); 7) microcalcification (MCAL); 8) round; 9) halo sign (AUR); and 10) up-location (UPL). These ten ultrasound indicators are based on internationally valid judgments of whether nodules are malignant tumors according to 2015ATA and EU-TIRADS. According to the number of 1) to 5) ultrasound features, the PTC patients were classified into 4A (one feature), 4B (two), 4C (three or four), and V (five). Their risk for PTC is 10, 50, 85, and 100%, respectively, which is the least upper bound of EU-TRADS. However, their accuracy needs further verification, which we intend to explore in further studies.

The following clinical features were recorded: birth date, sex, height, weight, T-nodule size (in cm), and the presence of antibodies to thyroid peroxidase (TPOAb), as may occur in Hashimoto’s disease. This disease manifests as an autoimmune attack on the thyroid. Clinical features were matched with five ultrasound characteristics, and the odds ratio and 95% confidence intervals were calculated for patients with a malignant single cancer.

### Statistics and Models

The dependent variable was malignancy, and the independent variables were the ultrasound features. The level of significance, at which the validity of independent variables was evaluated, was set at *p* ≤ 0.05. The odds ratio and 95% confidence interval were calculated for both training and test datasets.

Based on these characteristics, an equation indicating the probability of a malignant tumor was derived and applied to the data of the validation group. In PTC’s case, the test derivation prediction model (derivation set) was applied to the randomly selected 50% of patients, and the data of the remaining 50% provided the validation set. In FTC and MTC cases, all data were used for the prediction model.

The proposed models were assessed using a receiver–operating characteristic (ROC) curve, which is a plot of sensitivity against specificity. Sensitivity was defined as the proportion of positives identified correctly. Specificity was defined as the proportion of negatives identified correctly. The area under the ROC curve was calculated, and this provided the proportion of true results, including both the true positives and true negatives.

## Results

### Clinical Characteristics

This study included patients all of whom underwent ultrasound scanning. PTC in patients were confirmed by pathologic diagnosis after operation. The majority comprised female patients (79.6%). The age range of the patients was 15–74 years, with a mean age of 46.3 years and a median age of 47 years (standard deviation, 11.6 years).

### Association Between Ultrasound Characteristics of Nodules and Thyroid Cancers

A univariate analysis of ten potential ultrasound predictors of PTC in nodules is summarized in Table 1. Six characteristics showed statistically significant relationships (*p* < 0.05) with thyroid malignancy including ECHO (OR = 4.294), IRE (OR = 1.736), TTW (OR = 19.329), EF (OR = 1.953), ITC (OR = 4.672), and Round (0.080). TTW and ITC showed much higher OR levels than other characteristics. Neither odds ratio of CDFI, MCAL, AUR, or UPL has statistical significance (*p* > 0.05) in our patients.

**TABLE 1:** Association between ten ultrasound characteristics and PTC thyroid malignancy in the bivariate analysis (*n* = 1,385).

Features	Positive		Negative		OR	95%CI		χ^2^-mo	*p* Values
	Malignant	Benign	Malignant	Benign					
ECHO	958	324	42	61	4.294	2.842	7.961	53.074	3.213E-13
IRE	356	93	644	292	1.736	1.329	6.004	16.099	6.012E-05
TTW	797	65	203	320	19.329	14.205	24.312	464.049	6.32E-103
EF	584	161	416	224	1.953	1.539	6.368	30.086	4.133E-08
ITC	237	24	763	361	4.672	3.015	8.187	54.314	1.709E-13
CDFI	185	79	815	306	0.879	0.655	5.381	0.610	0.435
MCAL	147	69	853	316	0.789	0.577	5.323	1.954	0.162
Round	32	113	968	272	0.080	0.053	54.926	200.028	2.059E-45
AUR	16	6	984	379	1.027	0.399	3.246	0.034	0.854
UPL	248	97	752	288	0.979	0.747	5.491	0.007	0.934

The characteristics include the following: 1) hypo-echogenicity (ECHO); 2) irregular or micro-lobulated margins (IRE); 3) taller-than-wide ratio >1 (TTW); 4) echogenic focus (EF); 5) invasion of the thyroid capsule (ITC); 6) blood flow by color Doppler flow imaging (CDFI); 7) microcalcification (MCAL); 8) round; 9) halo sign (AUR); and 10) up-location (UPL).

In FTC patients, four characteristics showed statistically significant relationships (*p* < 0.05) with thyroid malignancy including ECHO (OR = 0.391), CDFI (OR = 4.734), MCAL (OR = 1.848), and AUR (OR = 13.399) (Table 2).

**TABLE 2:** Association between ten ultrasound characteristics and FTC thyroid malignancy in the bivariate analysis (*n* = 465).

Features	Positive		Negative		OR	95%CI		χ^2^-mo	*p* Values
	Malignant	Benign	Malignant	Benign					
ECHO	54	324	26	61	0.391	0.227	5.413	11.011	0.0009056
IRE	25	93	55	292	1.427	0.842	4.511	1.406	0.236
TTW	11	65	69	320	0.785	0.394	3.701	0.274	0.601
EF	36	161	44	224	1.138	0.701	4.556	0.160	0.689
ITC	7	24	73	361	1.442	0.599	3.276	0.330	0.566
CDFI	44	79	36	306	4.734	2.857	7.685	38.725	4.879E-10
MCAL	23	69	57	316	1.848	1.066	4.722	4.235	0.040
	22	113	58	272	0.913	0.533	4.265	0.039	0.844
AUR	14	6	66	379	13.399	4.971	13.482	37.114	1.114E-09
UPL	15	97	65	288	0.685	0.373	4.082	1.173	0.279

In MTC patients, six characteristics showed statistically significant relationships (*p* < 0.05) with thyroid malignancy including IRE (OR = 2.569), ITC (OR = 8.099), CDFI (OR = 4.281), ROUND (OR = 2.178), AUR (OR = 5.122), and UPL (OR = 2.195) (Table 3).

**TABLE 3:** Association between ten ultrasound characteristics and MTC thyroid malignancy in the bivariate analysis (*n* = 425).

Features	Positive		Negative		OR	95%CI		χ^2^-mo	*p* Values
	Malignant	Benign	Malignant	Benign					
ECHO	36	324	4	61	1.6944	0.58201	3.5822	0.557	0.455
IRE	18	93	22	292	2.5689	1.32085	4.911	7.114	0.008
TTW	9	65	31	320	1.4293	0.64959	3.5483	0.452	0.501
EF	21	161	19	224	1.5378	0.80054	4.1923	1.281	0.258
ITC	14	24	26	361	8.0994	3.7502	9.1357	33.380	7.581E-09
CDFI	21	79	19	306	4.2811	2.19495	6.4464	18.858	1.408E-05
MCAL	10	69	30	316	1.5266	0.71278	3.7	0.777	0.378
	19	113	21	272	2.1778	1.12763	4.63	4.759	0.029
AUR	3	6	37	379	5.1216	1.23001	4.1934	3.638	0.056
UPL	17	97	23	288	2.1945	1.12544	4.581	4.682	0.030

### Relationship Between Nodule Size and Ultrasound Characteristics in PTC

**FIGURE 1:**
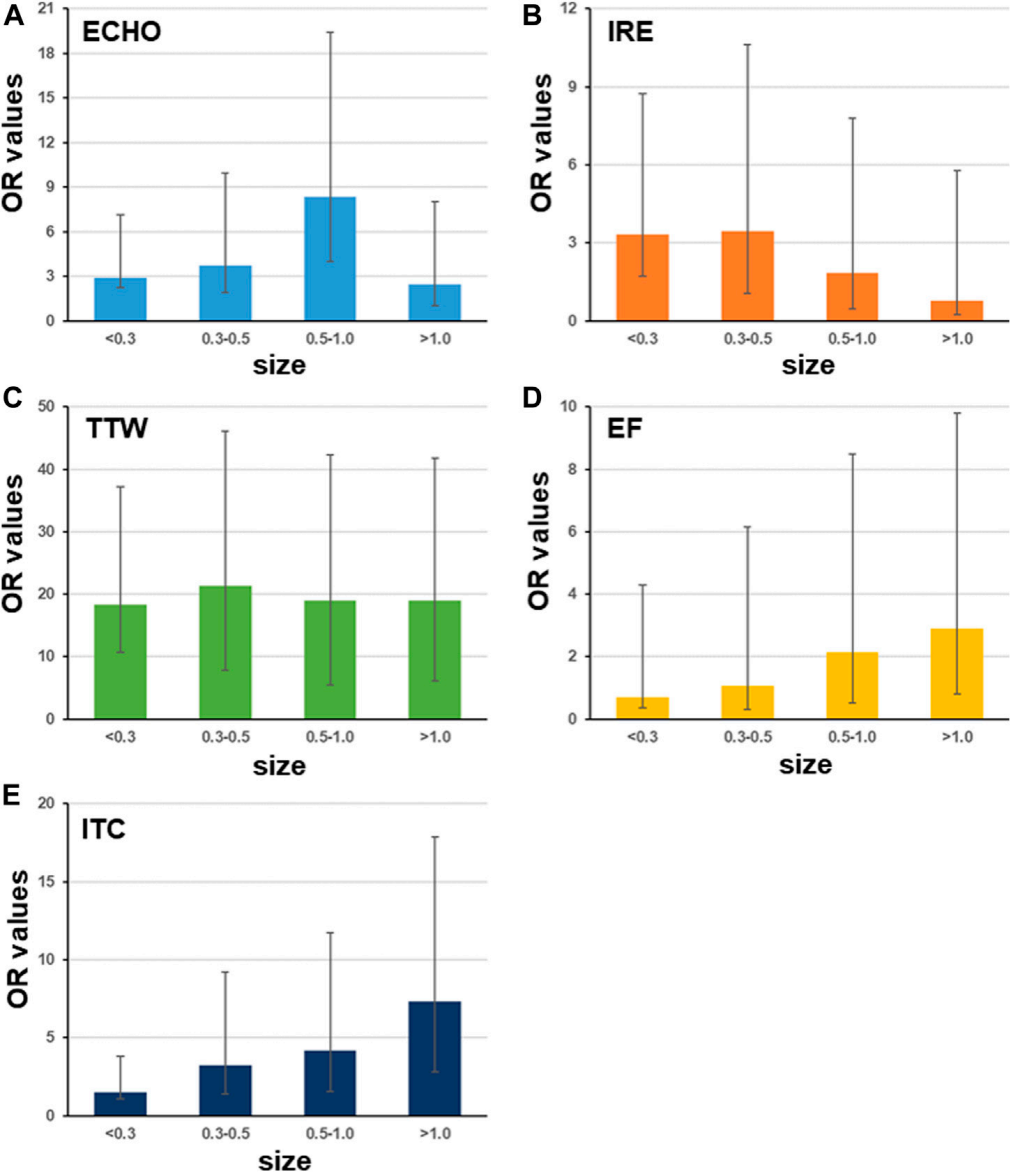
Odds ratio of ultrasound characteristics differs with nodule size. The patients are classified into groups with different nodule sizes including <0.3 cm, 0.3–0.5 cm, 0.5–1 cm, and >1 cm (sample size is 33, 187, 499, and 281, respectively). Among five ultrasound characteristics, only four have OR values with significance < 0.05. Bars indicate the 95%CI.

Small nodule size could lead to troubles in ultrasound image judgment and FNAB. Hence, it could be an effective factor for diagnosis and operation in clinical examination. The OR values of five ultrasound characteristics in patients with different nodule sizes were counted. The patients were classified into four groups according to their nodule size being < 0.3, 0.3–0.5, 0.5–1, and > 1 cm, respectively. Results displayed that ECHO had a dynamic OR values in all four groups with a maximum in the 0.5–1 cm group and a minimum in the > 1 cm group (Figure 1A). IRE had a decreasing pattern with increasing nodule size (Figure 1B). Four groups had similar OR values for TTW (Figure 1C). EF and ITC had an increased tendency with maximum OR values in the > 1 cm group (Figures 1D,E).

### Prediction Model and ROC Curves for PTC

The clinical predictive model of PTC in the thyroid nodule expressed the relationship between the probability of malignancy and the identified ultrasound characteristics, as follows:

1) Probability of PTC $=e^{x}/\left(1+e^{x}\right)$ , where *e* is the base of natural logarithms;
2) x=−3.474+1.899×ECHO+1.252×IRE+2.810×TTW+0.901×EF+2.791×ITC , where the presence of symptoms was scored as 1 if the patient had related symptoms and 0 if not. The statistical results are shown in Tables 3, 4.

**TABLE 4:** Results of the logistic regression analysis to determine the association between ultrasound characteristics and PTC malignancy.

Coefficients	Estimate	Std.	z	Pr(>|z|)
(Intercept)	−3.474	0.517	−6.716	1.860E-11
ECHO	1.899	0.489	3.883	1.030E-04
IRE	1.252	0.276	4.534	5.790E-06
TTW	2.810	0.240	11.731	2.000E-16
EF	0.901	0.236	3.824	1.320E-04
ITC	2.791	0.436	6.397	1.580E-10

**FIGURE 2:**
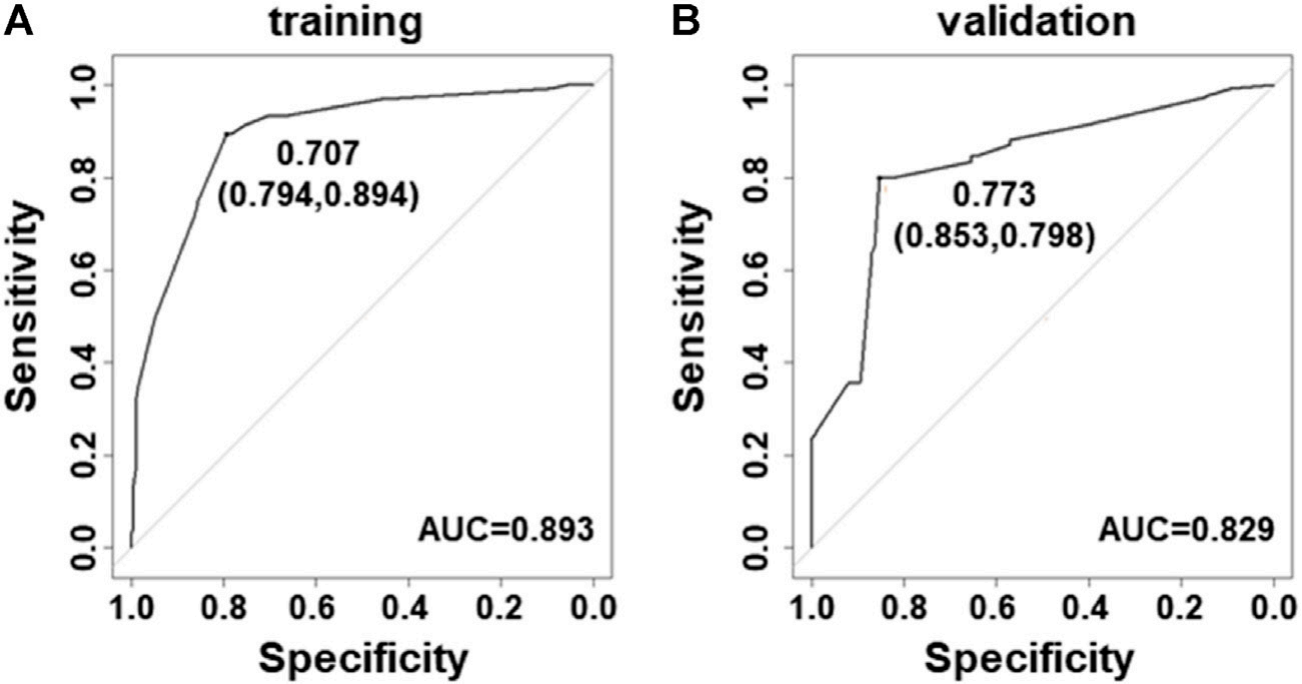
Receiver–operating characteristic curves for the prediction model in PTC. (A) Curve of the model derived from modeling data (AUC = 0.893). (B) Curve of the model from validated data (AUC = 0.829).

Next, the ROC curves were developed for modeling and validation datasets. The benign and malignancy could be distinguished by the prediction models. The area under the ROC curves (AUC) was 0.893 in datasets for model construction (Figure 2A), and 0.829 in the validation dataset (Figure 2B).

### Prediction Model and ROC Curves for FTC and MTC

The clinical predictive model of FTC and MTC in the thyroid nodule expressed the relationship between the probability of malignancy and the identified ultrasound characteristics, as follows:

3) Probability of FTC $=e^{x}/\left(1+e^{x}\right)$ , where *e* is the base of natural logarithms;
4) x=−2.461+0.787×IRE+1.433×CDFI+2.812×AUR , where the presence of symptoms was scored as 1 if the patient had related symptoms and 0 if not.
5) Probability of MTC $=e^{x}/\left(1+e^{x}\right)$ , where *e* is the base of natural logarithms;
6) x=−3.634+0.860×IRE+1.763×ITC+1.553×CDFI+0.695×UPL . The statistical results are shown in Tables 5, 6.

**TABLE 5:** Results of the logistic regression analysis to determine the association between ultrasound characteristics and FTC malignancy.

Coefficients	Estimate	Std.	z	Pr(>|z|)
(Intercept)	−2.461	0.218	−11.274	2.000E-16
IRE	0.787	0.296	2.656	7.900E-03
CDFI	1.433	0.279	5.145	2.670E-07
AUR	2.182	0.542	4.023	5.740E-05

**TABLE 6:** Results of the logistic regression analysis to determine the association between ultrasound characteristics and mTC malignancy.

Coefficients	Estimate	Std.	Error	Pr(>|z|)
(Intercept)	−3.634	0.352	−10.327	2.00E-16
IRE	0.860	0.394	2.184	2.890E-02
ITC	1.763	0.438	4.029	5.600E-05
CDFI	1.553	0.376	4.136	3.530E-05
UPL	0.696	0.375	1.856	6.350E-02

**FIGURE 3:**
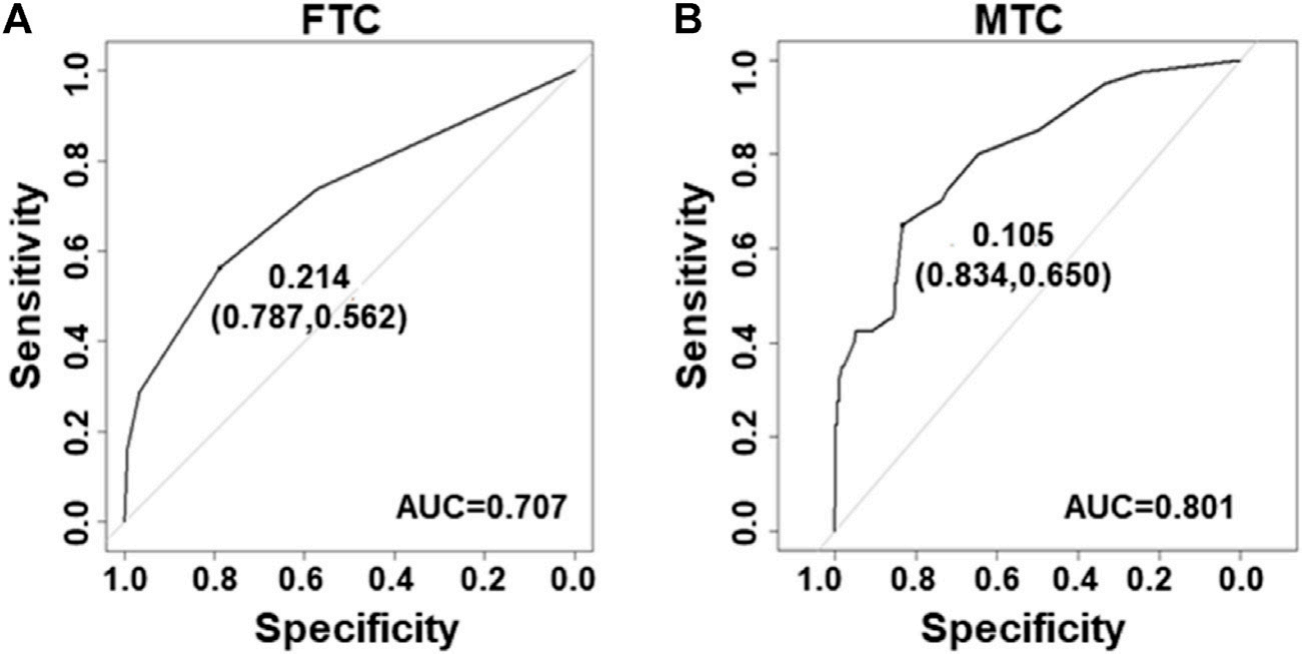
Receiver–operating characteristic curves for the prediction model in FTC and MTC. **(A)** Curve of the model derived from FTC data (AUC = 0.707). **(B)** Curve of the model from MTC data (AUC = 0.801).

Next, the ROC curves were developed for the FTC and MTC datasets. The area under the ROC curves (AUC) was 0.707 in datasets for FTC (Figure 3A) and 0.801 for MTC (Figure 3B).

### Models Revealed Different Probability in 4b/c Types Thyroid Nodules Between PTC and Benign Cases

**FIGURE 4:**
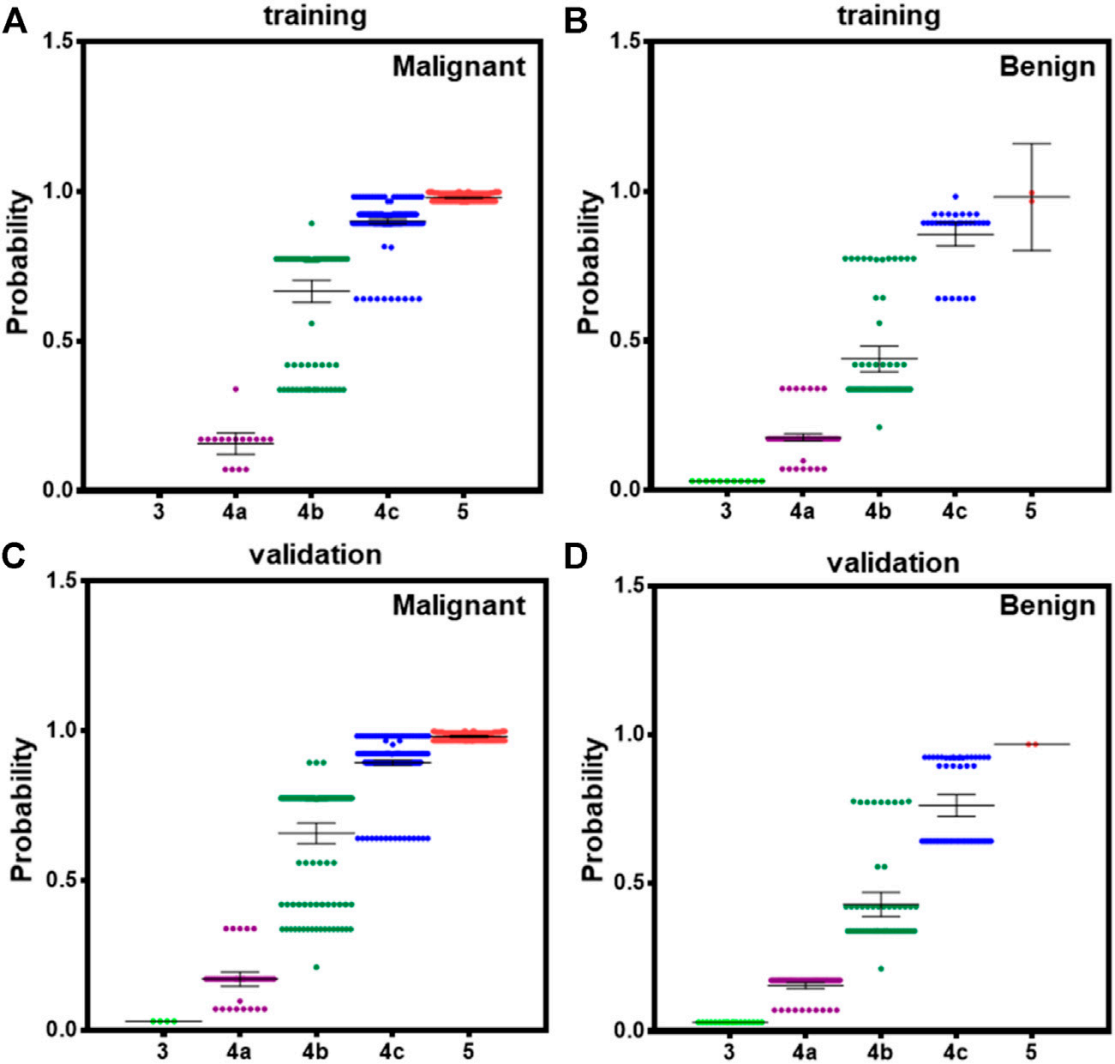
The averaged probability of malignancy predicted by models in different category malignant nodules according to TI-RADS. **(A)** Malignant cases derived from modeling data, **(B)** benign cases form modeling data, **(C)** malignant cases derived from validation data, and **(D)** benign cases form validation data. Bars indicate the 95%CI.

According to 2015ATA, nodules with ECHO plus one or more of ultrasound risk features are high suspicion malignancy (70–90%). Both BI-RADS and TI-RADS classified thyroid nodules into 0–6° according the number of positive ultrasound features. However, the combination of ultrasound features was not considered in the assessment of the probability. We calculated the probability in all PTC and benign nodules according to our model, and showed the average in 3–5 TI-RADS types. In the training dataset, malignant 4b nodules have significantly higher probability than benign 4b nodules, and malignant 4c nodules have a small difference with benign 4c nodules (Figures 4A,B). In validation datasets, both 4b and 4c type malignant nodules have significantly higher probability than benign nodules (Figures 4C,D). These results suggest that our module could better indicate the risk of PTC than both BI-RADS and TI-RADS.

## Discussion

In this study, five typical ultrasound characteristics were analyzed as predictor of thyroid malignancy. It was congruent with the findings of most previous studies (Moon et al., 2008; Wettasinghe et al., 2019; Alam et al., 2014). ECHO nodules showed a sensitivity of 87.1% in another study conducted on 500 patients (Papini et al., 2002). Previous studies suggest that marked hypo-echogenicity is commonly associated with benign tumors and rarely observed in cases of malignancy (Nachiappan et al., 2014; Kim et al., 2002). However, using only ECHO as malignancy characteristics showed only low specificity. It is different with a previous study in which Remonti et al., (2015) reported that the absence of elasticity, with a sensitivity of 87.9% and a specificity of 86.2%, achieved the best diagnostic accuracy in a study of 52 patients (Remonti et al., 2015). In patients with 0.5–1.0 cm nodule size, ECHO has much bigger OR values than in other nodule sizes (Figure 1A). Next, the incidence of IRE is proportional to the risk of malignancy (Papini et al., 2002; Remonti et al., 2015). In our results, nodules with a small size (<0.5 cm) has higher OD of IRE than big nodules (Figure 1B). It suggested that IRE is more valuable for prediction of PTC in small nodules. Another study indicated that a taller-than-wide shape is very specific for distinguishing malignant from benign thyroid nodules in both unilateral and bilateral cancer (Moon et al., 2008). In this study, we found that OR values of TTW did not vary in nodules with different sizes (Figure 1C). It suggested that the nodule size has a limit effect on OR values of TTW. Previous studies suggested that EF and ITC are reliable indicators of diagnostic accuracy (Wettasinghe et al., 2019) (Nachiappan et al., 2014). Our results showed that both EF and ITC have biggest OR values in nodules with size >1 cm. In general, while using ECHO, IRE, EF, and ITC to assess risk of PTC, one should also consider the nodule size; then none of these five features would be a risk factor for FTC and MTC according to OD values (Table 4). Instead of them, IRE, CDFI, and AUR have significant high OD values for FTC. In MTC’s case, IRE, ITC, CDFI, and UPL are significant risk factors. Here, CDFI, AUR, and UPL are not risk factors for PTC.

Next, integration of five ultrasound characteristics to predict thyroid malignancy is more reliable than only one. We assessed individual ultrasound characteristics separately to counteract the overlap that occurs when more than one ultrasound characteristic is considered (Frates et al., 2006). A multivariate model is more appropriate. We developed logistic models for prediction of PTC. The model could increase the accuracy to 89 and 83% in modeling and validation datasets, respectively (Figure 2), and this might be due to the fact that the model considered the weight factor of ultrasound features and the combination pattern. In general, our model could more easily give a reliable prediction than using only one ultrasound characteristic. Also, logistic models for prediction of FTC and MTC were built with an accuracy of 70 and 80%, respectively (Figure 3).

In addition, definition of high-risk category (4b and 4c, 10–85%) following 2015ATA did not consider the detail number of ultrasound features, whereas our result clearly revealed that malignancy and benign nodules with two or three of ultrasound features have significantly different risk probability based on our logistical model (Figure 4). This could facilitate the clinical performance to convince patients for FNAB and operation.

In conclusion, it is difficult to predict the malignancy of thyroid nodules using ultrasound features. Also applying ultrasound features to assess PTC is restricted by the nodule size. Here, our modified logistic model using ECHO, TTW, IRE, EF, and ITC could give more than 80% accuracy for predicting the PTC.

## Data Availability

The original contributions presented in the study are included in the article/Supplementary Material, further inquiries can be directed to the corresponding authors.
